# IRF4 is a novel mediator for neuronal survival in ischaemic stroke

**DOI:** 10.1038/cdd.2014.9

**Published:** 2014-02-07

**Authors:** S Guo, Z-Z Li, D-S Jiang, Y Y Lu, Y Liu, L Gao, S-M Zhang, H Lei, L-H Zhu, X-D Zhang, D-P Liu, H Li

**Affiliations:** 1 Department of Cardiology, Renmin Hospital of Wuhan University, Cardiovascular Research Institute, Wuhan University, Wuhan, China; 2 Cardiovascular Research Institute, Wuhan University, Wuhan, China; 3 State Key Laboratory of Medical Molecular Biology, Department of Biochemistry and Molecular Biology, Institute of Basic Medical Sciences, Chinese Academy of Medical Sciences and Peking Union Medical College, Beijing, China; 4 College of Life Sciences, Wuhan University, Wuhan, China; 5 Department of Cardiology, Institute of Cardiovascular Disease, Union Hospital, Tongji Medical College, Huazhong University of Science and Technology, Wuhan, China; 6 Wuhan Center for Magnetic Resonance, State Key Laboratory of Magnetic Resonance and Atomic and Molecular Physics, Wuhan Institute of Physics and Mathematics, Chinese Academy of Sciences, Wuhan, China

**Keywords:** IRF4, SRF, ischaemic stroke, neuronal survival

## Abstract

Neuroprotection following ischaemic stroke is driven by the interplay between regulatory transcription factors and endogenous protective factors. IRF4, a member of the interferon regulatory factor (IRF) family, is implicated in the survival of tumour cells. However, its role in the survival of normal cells including neurons remains elusive. Using genetic approaches, we established a central role for IRF4 in protection against ischaemia/reperfusion (I/R)-induced neuronal death. IRF4 was expressed in neurons, and induced by ischaemic stroke. Neuron-specific IRF4 transgenic (IRF4-TG) mice exhibited reduced infarct lesions, and this effect was reversed in IRF4-knockout mice. Notably, we revealed that IRF4 rescues neurons from I/R-induced death both *in vivo* and *in vitro*. Integrative transcriptional and cell survival analyses showed that IRF4 functions mechanistically as a transcription activator of serum response factor (SRF) crucial to salvage neurons during stroke. Indeed, the expression of SRF and SRF-dependent molecules was significantly upregulated upon IRF4 overexpression and conversely inhibited upon IRF4 ablation. Similar results were observed in oxygen glucose deprivation (OGD)-treated primary cortical neurons. Furthermore, we identified the IRF4-binding site in the promoter region of the *SRF* gene essential for its transcription. To verify the IRF4–SRF axis *in vivo*, we generated neuron-specific SRF knockout mice, in which SRF exerted profound cerebroprotective effects similar to those of IRF4. More importantly, the phenotype observed in IRF4-TG mice was completely reversed by SRF ablation. Thus, we have shown that the IRF4–SRF axis is a novel signalling pathway critical for neuronal survival in the setting of ischaemic stroke.

Stroke is the most severe and devastating neurological disease globally. In addition, stroke is the leading cause of permanent disability in adults and poses a significant threat to the quality of life.^1, 2^ Ischaemic stroke is the predominant stroke type, in most cases attributable to the interception of the middle cerebral artery (MAC).^3^ To date, recombinant tissue plasminogen activator remains the principal drug approved for pharmacological intervention to salvage the brain. Nevertheless, tissue damage occurs even after recanalisation of the occluded arteries.^4^ As a result, despite the current failure in clinical trials, neuroprotection remains the central focus of acute stroke treatment after reperfusion. Although there are likely many contributors to these unsuccessful clinical therapies, the current neuroprotective drugs selected for stroke may not be the most efficacious.^5^ Neuroprotection involves the tight regulation of endogenous defensive signalling pathways, many of which converge on transcription factors in the nucleus, namely, CREB, Nrf2 and HIF1.^6^ Moreover, although implicated in neuronal survival, the knowledge of the biological role of several factors, such as serum response factor (SRF), remains rudimentary *in vivo.*^7^ Thus, there is great interest in identifying novel protective transcription factors for therapeutic interventions for stroke.

Interferon (IFN) regulatory factors (IRFs), consisting of nine members, were originally identified as transcriptional regulators of type I IFNs. IRF4, also known as multiple myeloma oncogene-1, was recognised as a key regulator of lymphoid, myeloid and dendritic cell differentiation.^8^ Moreover, the expression of IRF4 was initially asserted to be restricted to immune cell and melanocytic lineages.^9, 10^ Recently, we and others have indicated that IRFs exert additional functions in insulin resistance, cardiac pathology, cell survival and oncogenesis.^8, 9, 11, 12^ For example, we reported that the overexpression of IRF4-aggravated cardiac hypertrophy was mediated through the transcriptional regulation of CREB.^13^ We also showed that IRF4 expression was readily detectable in the brain, heart, liver and kidney, indicating more diverse biological functions of IRF4 than expected.^13^ In addition, the role of IRF4 in oncogenesis, particularly haematopoietic malignancies, has been well documented.^14, 15^ For example, IRF4 has been implicated in the survival of multiple myeloma cell lines.^16^ The functions of IRF4 in neurological diseases, however, have not been elucidated thus far.

In this study, we evaluated the neuronal effect of IRF4 in a stroke model. Interestingly, we observed significant upregulation of IRF4 upon stroke insults. Using neuron-specific IRF4 transgenic (IRF4-TG) and knockout (IRF4-KO) mice, we demonstrated that IRF4 is a potent neuroprotective factor that rescues neurons from ischaemia/reperfusion (I/R)-induced degeneration and apoptosis both *in vivo* and *in vitro*. Mechanistically, we revealed that IRF4 facilitated neuronal survival via inducing SRF expression. Using genetic manipulations, we further demonstrated that SRF is neuroprotective against stroke *in vivo*. Thus, our results demonstrated the IRF4–SRF axis to be a novel signalling pathway critical for neuronal survival in the setting of ischaemic stroke.

## Results

### IRF4 expression is induced in neurons after ischaemic stroke

**Figure 1:**
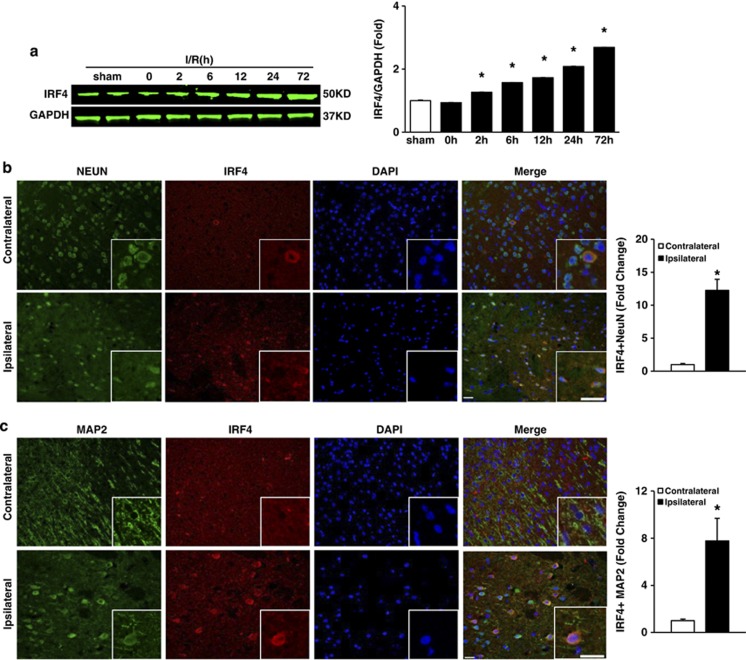

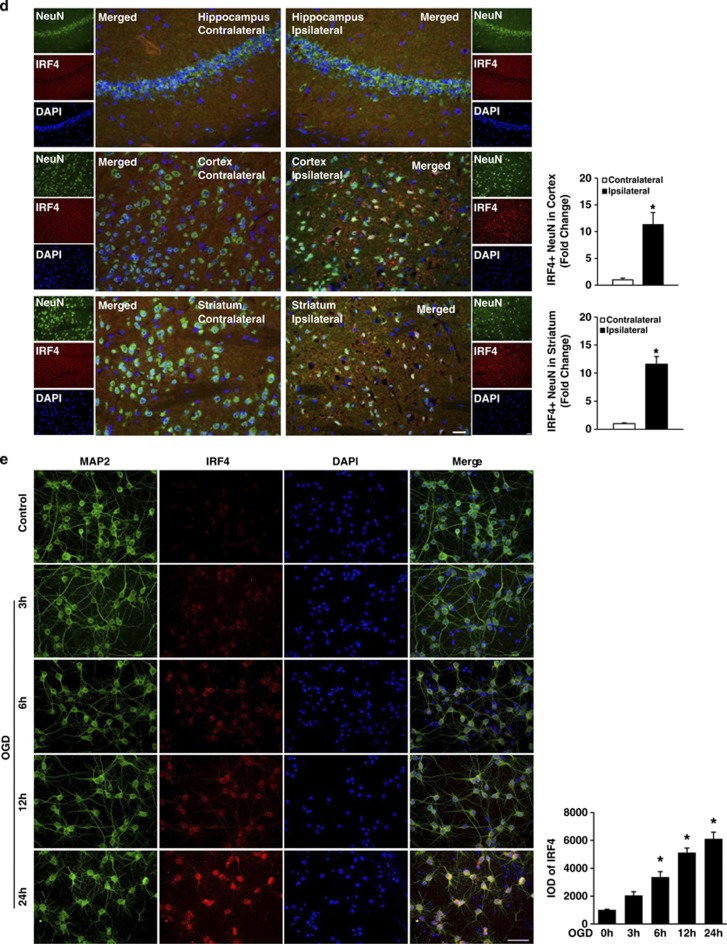
The expression of IRF4 is elevated during middle cerebral artery occlusion and oxygen glucose deprivation (OGD). (**a**) Immunoblotting of IRF4 in brains subjected to ischaemia/reperfusion (I/R) for the indicated time points. GAPDH served as a loading control in each lane. For each time point, three independent experiments were performed in triplicate. *P<0.0001 *versus* sham, *post hoc* Tukey's test with Bonferroni correction. (**b** and **c**) IRF4 is upregulated in neurons following stroke onset. The mice were subjected to I/R for 24 h, and their brains were co-stained with IRF4 (red) and either (**b**) NEUN (green) or (**c**) MAP2 (green). The nuclei were stained using 4′6-diamino-2-phenylindole (DAPI). The insets show higher magnification views. Scale bar: 20 *μ*m. Right panel: the number of IRF4-positive neurons was quantified. Four to five independent experiments were performed. *P=0.0063 (**b**) and 0.0240 (**c**) *versus* contralateral controls, unpaired Student's *t*-test. (**d**) Representative immunofluorescence images of IRF4 (red), NEUN (green) and DAPI (blue) in the hippocampus, cortex and striatum regions from the ipsilateral and contralateral sides, following 24-h I/R. Merged images are shown. Scale bar: 20 *μ*m. Right panels: the number of IRF4-positive neurons was quantified. Four independent experiments were performed. *P=0.0185 (cortex) and 0.0003 (striatum) *versus* contralateral controls unpaired Student's *t*-test. (**e**) The primary cortical neurons were treated with OGD/reperfusion for the indicated time points. The cells were then co-stained with IRF4 (red), MAP2 (green) and nuclei (DAPI). Scale bar: 50 *μ*m. Right panel shows IRF4 intensity in neurons. Three independent experiments were performed. *P=0.0004 (6 h), <0.0001 (12 h) and <0.0001 (24 h) *versus* control, *post hoc* Tukey's test with Bonferroni correction. The values are the means±S.E

To investigate whether IRF4 is involved in ischaemic stroke, we tested IRF4 expression in an established stroke mouse model using left middle cerebral occlusion (MCAO) for 45 min,^17^ followed by reperfusion ceded at the indicated time points (Figure 1a). Compared with the sham controls, the MCAO/reperfusion-treated hemisphere exhibited a time-dependent increase in IRF4 protein expression, as shown using immunoblotting (Figure 1a). Previous reports demonstrated that IRF4 expression was restricted to immune cells.^9, 10^ Unexpectedly, we observed that the ipsilateral cortex exhibited stronger IRF4 staining, which was co-expressed with neuron-specific nuclear protein (NeuN; Figure 1b) and microtubule-associated protein-2 (MAP2; Figure 1c) *in vivo*. Furthermore, immunostaining of IRF4 and NeuN demonstrated that neuronal IRF4 was specifically induced by I/R, as demonstrated by an increased number of IRF4-positive neurons in the ipsilateral cortex and striatum but not in the hippocampus (Figure 1d), where cerebral blood flow was not supplied by the MAC.^17^ This observation was further confirmed *in vitro*. The primary cortical neurons were isolated from the rats and subjected to oxygen-glucose deprivation (OGD) for 1 h, followed by normoxia culture for the indicated times to simulate I/R. In concert, neuronal IRF4 expression was elevated in a time-dependent manner after OGD/reperfusion (Figure 1e). Together, our data indicated that neuronal IRF4 may be involved in ischaemic stroke.

### Neuron-specific IRF4 deficiency aggravates ischaemic cerebral injury

**Figure 2:**
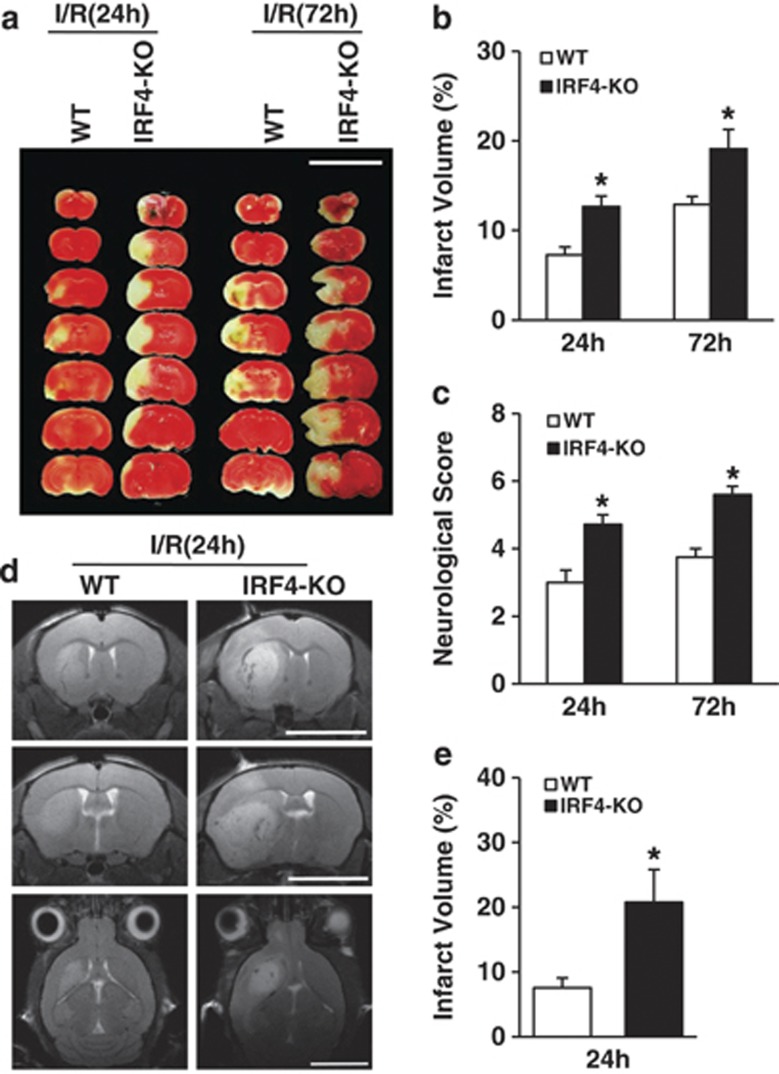
Depletion of IRF4 in neurons led to potentiated cerebral injury. (**a**–**c**) The mice were subjected to I/R for 24 or 72 h. (**a**) 2,3,5-Triphenyl-2H-tetrazolium chloride (TTC)-stained brains from WT and IRF4-KO mice at the indicated times after I/R. Scale bar: 10 mm. (**b**) Quantification of infarct volumes 24 and 72 h after I/R. Four to eight independent experiments were performed for each time point. (**c**) The mice were assessed for neurological deficit scores at the indicated time points. Four to eight independent experiments were performed for each time point. (**d**) Representative MRI images of live mice 24 h after I/R. Scale bar: 5 mm. (**e**) Quantification of infarct volumes using MRI. Four to five independent experiments were performed. *P=0.0046 (**b**, 24 h), 0.0460 (**b**, 72 h), 0.0032 (**c**, 24 h), 0.0012 (**c**, 72 h) and 0.0272 (**e**) compared with WT mice unpaired Student's *t*-test. The values are the means±S.E

To this end, we crossed Irf4^fl/fl^ mice with mice carrying a Cre recombinase driven by the neuron-specific promoter CaMKII*α* (Ca2+/calmodulin-dependent protein kinase II; Supplementary Figure 1a). The generated neuron-specific IRF4-KO (hereafter referred to as IRF4-KO) mice grew normally and were active and fertile. The genetic knockdown of IRF4 was verified using western blot analyses. Intriguingly, IRF4 expression in the brains of IRF4-KO mice was largely reduced (Supplementary Figure 1b), indicating that neurons may be a primary target of IRF4 in central nervous system. Indeed, only a few IRF4-positive microglia can be detected in the brains (Supplementary Figure 2). We then determined whether IRF4 deficiency has a direct impact on the integrity of the cerebral vasculature. Indian ink staining showed no gross anatomical differences in the Circle of Willis, anterior cerebral artery, MCAs and posterior arteries between IRF4-KO and wild-type (WT; CaMKII*α*-Cre) mice (Supplementary Figure 3). Because hypertension and arrhythmia are particularly profound stroke risk factors, we also inspected the blood pressure, heart rate and blood gases.^1^ No significant differences were noticed between the IRF4-KO and WT mice (Supplementary Table 1). The mice were subjected to MCAO for 45 min, followed by reperfusion ceded at 24 or 72 h after MCAO. Interestingly, 2,3,5-triphenyl-2H-tetrazolium chloride staining (Figure 2a) and quantification (Figure 2b) revealed that in response to I/R for 24 or 72 h, the IRF4-KO mice displayed approximately 74.27% and 48.40% enlarged infarct volumes, respectively, compared with their WT littermates (Figure 2b). Consequently, the neurological deficits were also more severe in the IRF4-KO mice, as shown by approximately 57.14% and 49.33% increases in the neurological scores compared with the WT mice at both time points, respectively (Figure 2c). We next validated these observations in live mice, and the IRF4-KO mice displayed an approximately 1.7-fold increase in infarct volume, as shown using magnetic resonance imaging (MRI) and quantification (Figures 2d and e). Together, these results indicated that neuron-specific IRF4 deletion deteriorates the stroke outcomes.

### Neuron-specific IRF4 transgene is cerebroprotective

**Figure 3:**
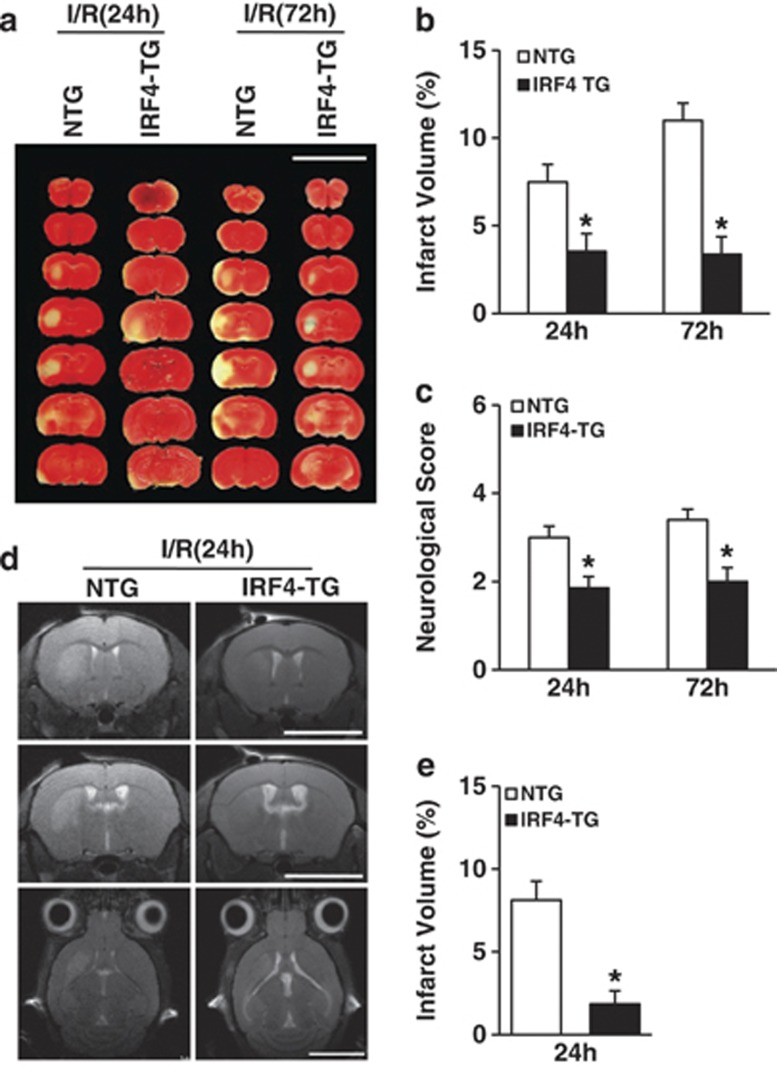
Neuron-specific IRF4 overexpression is cerebroprotective during stroke. (**a** and **b**) The mice were subjected to I/R for 24 or 72 h. (**a**) TTC-stained brains from NTG and IRF4-TG mice at the indicated times after I/R. Scale bar: 10 mm. (**b**) Quantification of infarct volumes 24 and 72 h after I/R. Five to seven independent experiments were performed for each time point. (**c**) The mice were assessed for neurological deficit scores at the indicated time points. Five to seven independent experiments were performed for each time point. (**d**) Representative MRI images of live mice 24 h after I/R. Scale bar: 5mm. (**e**) Quantification of infarct volumes using MRI. Three to five independent experiments were performed. *P=0.0066 (**b**, 24 h), 0.0007 (**b**, 72 h), 0.0103 (**c**, 24 h), 0.0081 (**c**, 72 h) and 0.0086 (**e**) compared with NTG mice unpaired Student's *t*-test. The values are the means±S.E

After elucidating that the inhibition of neuronal IRF4 induction may be detrimental, we thus hypothesised that the forced overexpression of IRF4 in neurons may protect against cerebral injury. To address this issue, we crossed Irf4-floxed mice with CaMKII*α*-Cre mice to generate neuron-specific IRF4-TG mice (hereafter referred to as IRF4-TG; Supplementary Figure 4a). The validation of IRF4-TG mice was shown using western blot analysis (Supplementary Figure 4b). Similarly, no differences in the integrity of the cerebral vasculature were noted (Supplementary Figure 3), and no differences were observed in the blood pressure, heart rate and blood gases (Supplementary Table 1). However, we observed approximately 52.71% and 69.36% reductions in the infarct volume in the IRF4-TG mice after 24 or 72 h of I/R, respectively, compared with their non-transgenic (NTG) littermates (Figures 3a and b). Moreover, neurological function was ameliorated upon IRF4 overexpression (Figure 3c). In concert, the infarct size was dramatically diminished in the live IRF4-TG mice (reduced by 77.43% compared with the NTG mice; Figures 3d and e). Taken together, these data demonstrated that neuronal IRF4 provides cerebroprotection against stroke insults.

### IRF4 provides neuroprotection in cerebral ischaemia *in vivo*

**Figure 4:**
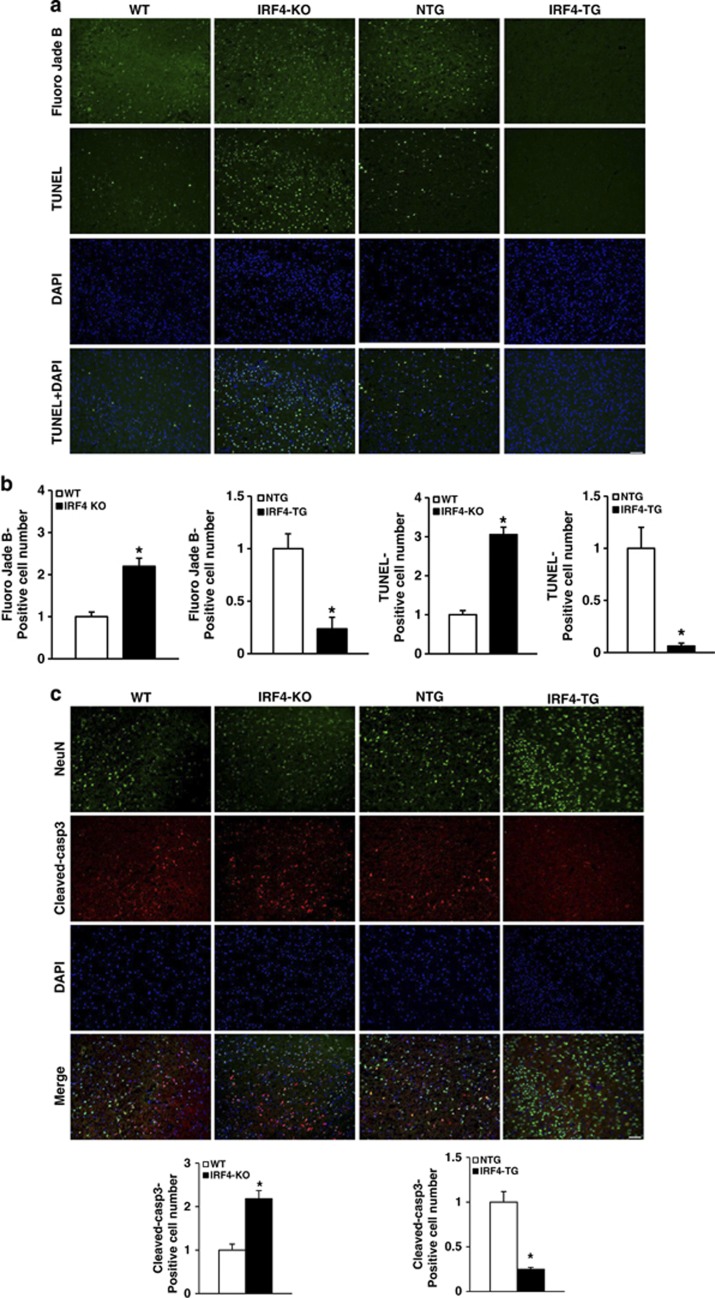

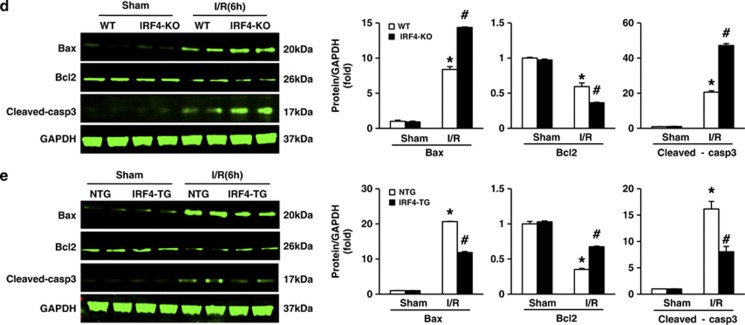
(Continued)

Neurons are particularly vulnerable compared with other cell types in the brain.^18^ Considering the profound effects of IRF4 on cerebroprotection, we thus determined whether IRF4 directly impacted neuronal death *in vivo*. Brain cryosections 24 h after I/R were prepared with Fluoro-Jade B staining for neuronal death and were analysed with the terminal deoxynucleotidyl transferase-mediated dUTP-biotin nick end labelling (TUNEL) assay for apoptosis (Figure 4a). The number of Fluoro-Jade B-positive cells increased by 120.01% in the IRF4-KO mice; conversely, the number of Fluoro-Jade B-positive cells decreased by 76.48% in IRF4-TG compared with their control littermates (Figure 4b). Similarly, a 205.37% increase and 93.80% decrease in the number of TUNEL-positive cells were observed in the IRF4-KO and IRF4-TG mice, respectively (Figure 4b). In accordance, the protective effect of IRF4 on neuroapoptosis was further verified via cleaved-caspase-3 staining *in vivo* (Figure 4c). Furthermore, western blotting showed that although I/R for 6 h elevated the expression of pro-apoptotic Bax and cleaved-caspase-3 and reduced the expression of anti-apoptotic Bcl2, these observations were significantly abrogated in IRF4-TG mice and were further enhanced in IRF4-KO mice (Figures 4d and e). Together, our data provided a causal link between IRF4 and neuronal survival in ischaemic cerebral injury.

### IRF4 rescues neurons from OGD-induced death *in vitro*

**Figure 5:**
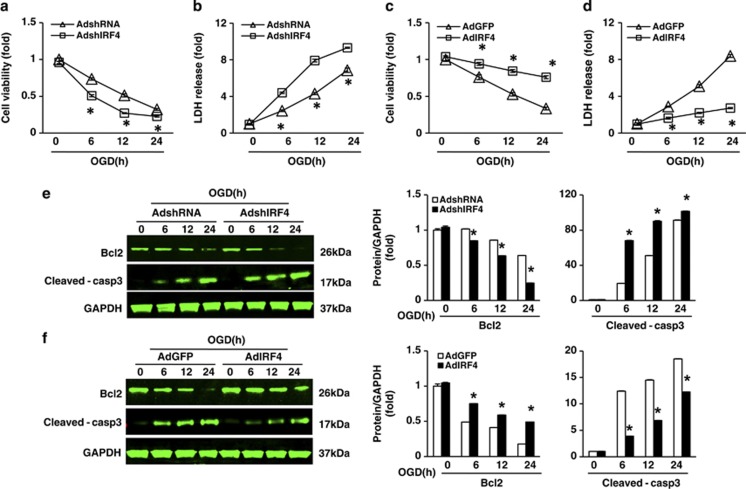
IRF4 protects neurons from OGD-induced cell death. (**a**–**d**) Quantification of neuronal death following OGD at the indicated time points by cell viability (**a**, **c**) and LDH release (**b**, **d**). The primary neurons were infected with AdshIRF4 (**a**, **b**), AdIRF4 (**c**, **d**) or control adenoviruses. For each time point, three independent experiments were performed in triplicate. *P<0.0001 (**a**, **b**) compared with AdshRNA, unpaired Student's *t*-test; *P=0.0004 (**c**, 6 h), <0.0001 (**c**, 12 and 24 h) and <0.0001 (**d**) compared with AdGFP, unpaired Student's *t*-test. (**e** and **f**) Immunoblotting of Bcl2 and cleaved caspase-3 in primary neurons infected with AdshIRF4 (**e**) or AdIRF4 (**f**) before OGD for the indicated times. GAPDH served as a loading control in each lane. The right panels show the quantification of the indicated protein levels normalised to GAPDH. For each time point, three independent experiments were performed in triplicate. *P<0.0001 *versus* AdshRNA in **e**, unpaired Student's *t*-test; *P<0.0001 *versus* AdGFP in **f**, unpaired Student's *t*-test. The values are the means±S.E

To test the direct role of IRF4 in neuronal cell fate, we next isolated rat primary cortical neurons and infected them with adenovirus harbouring either WT IRF4 (AdIRF4) or short-hairpin IRF4 (AdshIRF4).^13^ The cultured neurons were treated with OGD for 60 min, followed by normal medium for the indicated durations to mimic I/R *in vitro*. Accordingly, AdshIRF4 rendered primary neuronal cultures more prone to death when challenged by OGD, as demonstrated by the inhibited cell viability and enhanced lactate dehydrogenase (LDH) release (Figures 5a and b). Similarly, AdIRF4 rescued primary cortical neurons from an OGD-induced demise (Figures 5c and d). Western blot analyses also showed an effect of IRF4 on apoptotic programmes that was similar to that observed *in vivo* (Figures 5e and f). Collectively, our previous data indicated that IRF4 is necessary for neuronal survival after I/R insults.

### IRF4 mediates neuronal survival by preserving the SRF expression levels

**Figure 6:**
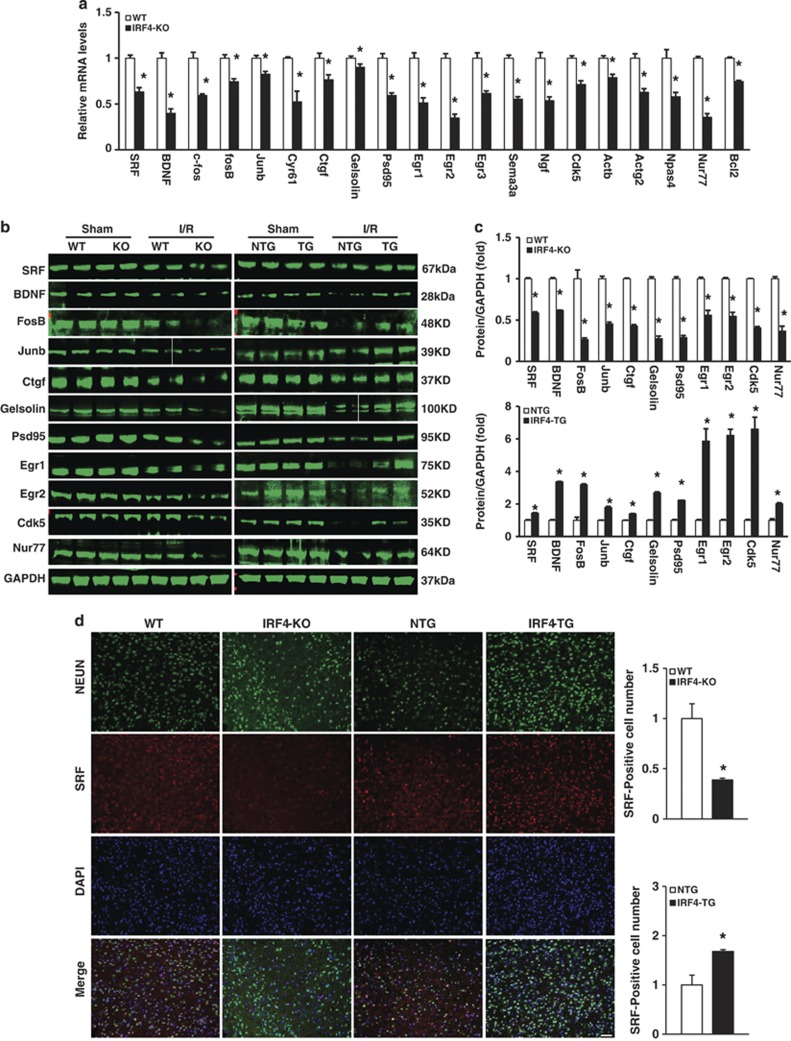

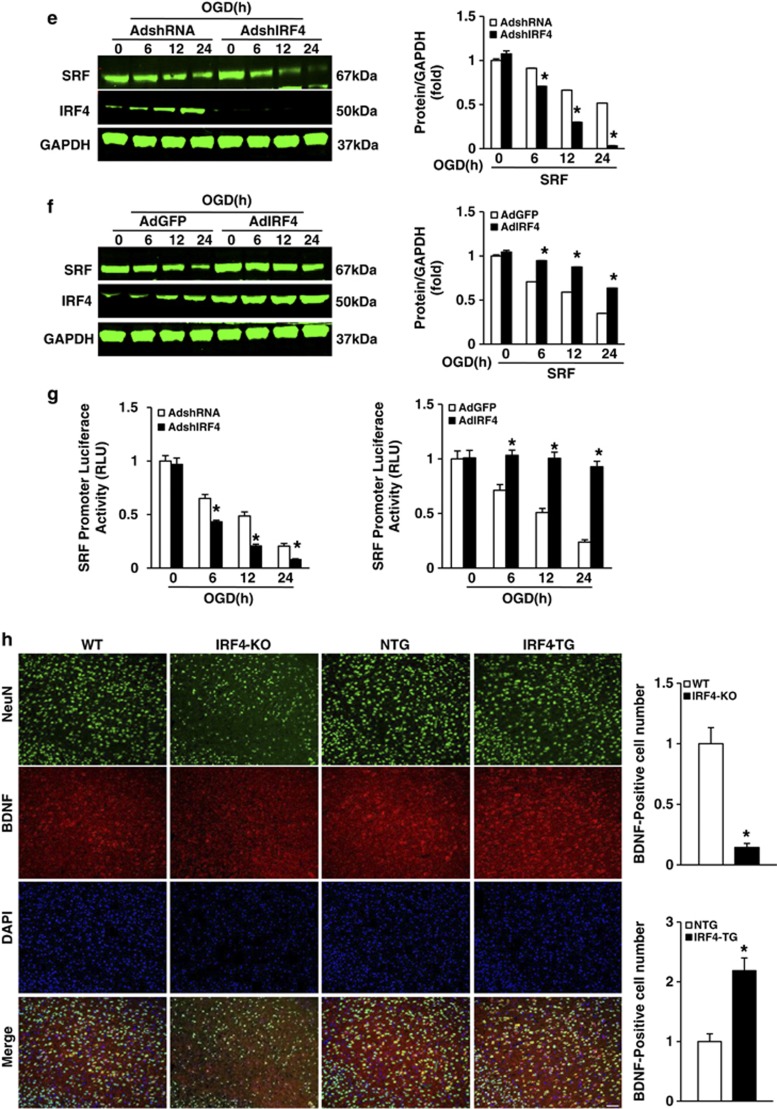

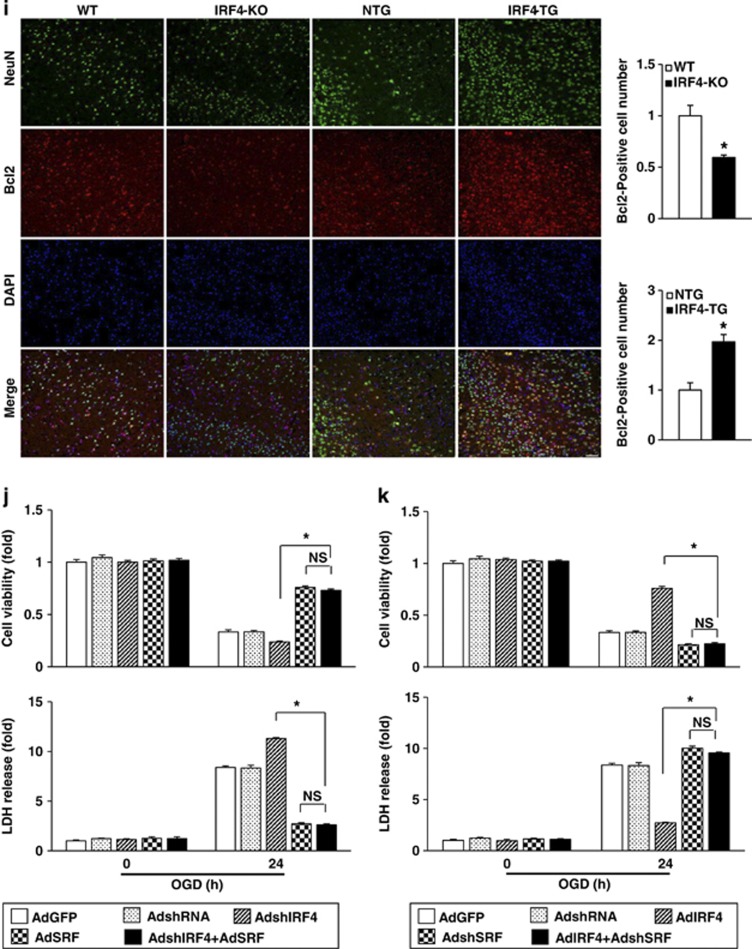
(Continued)

Given the profound effects of both the inhibition and overexpression of IRF4 on neuronal cell fate in the setting of I/R, we next sought to determine the molecular basis of this phenomenon. A genome-wide microarray screening revealed that the mRNA levels of a myriad of pro-survival factors, such as brain-derived neurotrophic factor (BDNF), FosB and Egr1, were significantly reduced in the IRF4-KO mice following I/R (data not shown).^19, 20, 21, 22^ Serum-response factor (SRF), a ubiquitous transcription factor upstream of these factors that binds to the *cis*-acting SRE, thus activates SRE-mediated gene expression.^23^ Moreover, SRF is required for the neuroprotection of cortical neurons by BDNF.^7^ However, whether SRF protects neurons during stroke remains undefined. Interestingly, the mRNA level of SRF along with that of SRF-dependant factors was significantly lower in the IRF4-KO mice compared with the WT controls (Figure 6a). Western blot analysis further showed that after I/R, SRF-dependent pathways were significantly inhibited in mouse brains (Figures 6b and c). IRF4-TG, however, restored the expression of SRF and its downstream proteins; in sharp contrast, IRF4-KO further diminished the expression of SRF-dependent proteins (Figures 6b and c). In addition, the co-staining of SRF and NeuN showed that IRF4-KO mice exhibited less SRF-positive neurons than WT mice (61.13% reduction), whereas IRF4-TG mice preserved neuronal SRF expression (67.85% increase compared with NTG; Figure 6d). The positive correlation between IRF4 and SRF protein expression was further confirmed in the *in vitro* paradigm (Figures 6e and f), although the causal role of IRF4 remains unclear. Given that IRF family members are well-characterised transcription factors, we speculated that IRF4 may positively regulate SRF transcription.^8^ Hence, we next tested SRF luciferase activity after OGD in the presence or absence of AdshIRF4 and AdIRF4 (Figure 6g). AdshIRF4 increasingly inhibited SRF promoter transcription after OGD, which was largely preserved by Ad-IRF4 (Figure 6g). The IRFs recognises a core 5′-GAAA-3′ sequence termed the IFN-stimulated response element in the promoter region of downstream genes.^8^ Using a bioinformatics approach, we only identified one putative IRF4-binding sites in the 1800-bp promoter region of murine SRF. To validate the importance of this binding site, the 5′-GAAA-3′ sequence in the P3 region was mutated to generate mutant murine SRF promoter (Mu-mSRF-Luc). As expected, IRF4 retained the ability to increase the promoter activity of WT (WT-mSRF-Luc) but not mutant SRF promoter (Mu-mSRF-Luc; Supplementary Figure 5a), underscoring the importance and necessity of this binding site. Next, we determined whether IRF4 directly binds to the P3 region. Four adjacent regions (P1, P2, P4 and P5) without the putative 5′-GAAA-3′ binding motif were selected and served as negative controls. Chromatin immunoprecipitation (ChIP) of IRF4 was performed followed by quantitative PCR of the murine SRF promoter. Interestingly, IRF4 ChIPs were enriched for the P3 region but not any other regions (Supplementary Figure S5b). Thus, the P3 region contains the primary site for direct IRF4 binding. In accordance, the IRF4-KO mice had significantly fewer BDNF- and Bcl2-positive neurons; these neurons were well preserved in the IRF4-TG mice (Figures 6h and i). Next, we asked whether the IRF4–SRF axis contributes to IRF4-mediated neuronal survival. We employed an adenovirus harbouring SRF (Ad-SRF) or an SRF short-hairpin (Ad-shSRF). Intriguingly, although AdshIRF4 increased the vulnerability of OGD-challenged neurons, this effect was completely negated when these neurons were co-infected with Ad-SRF (Figure 6j). Notably, the salvage of neurons by the co-infection was comparable with that of AdSRF infection alone (Figure 6j), indicating that SRF functions downstream of IRF4. Conversely, the co-infection of Ad-IRF4 and Ad-shSRF completely reversed the IRF4-mediated neuroprotection (Figure 6k). Similarly, no significant difference was noted between Ad-shSRF and the co-infection group, suggesting that SRF fully recapitulated the IRF4-mediated pro-survival effect. Taken together, we demonstrated that the salvage of neurons by IRF4 is directly regulated by the preservation of SRF transcription in neurons.

### IRF4-mediated neuroprotection is SRF dependent

**Figure 7:**
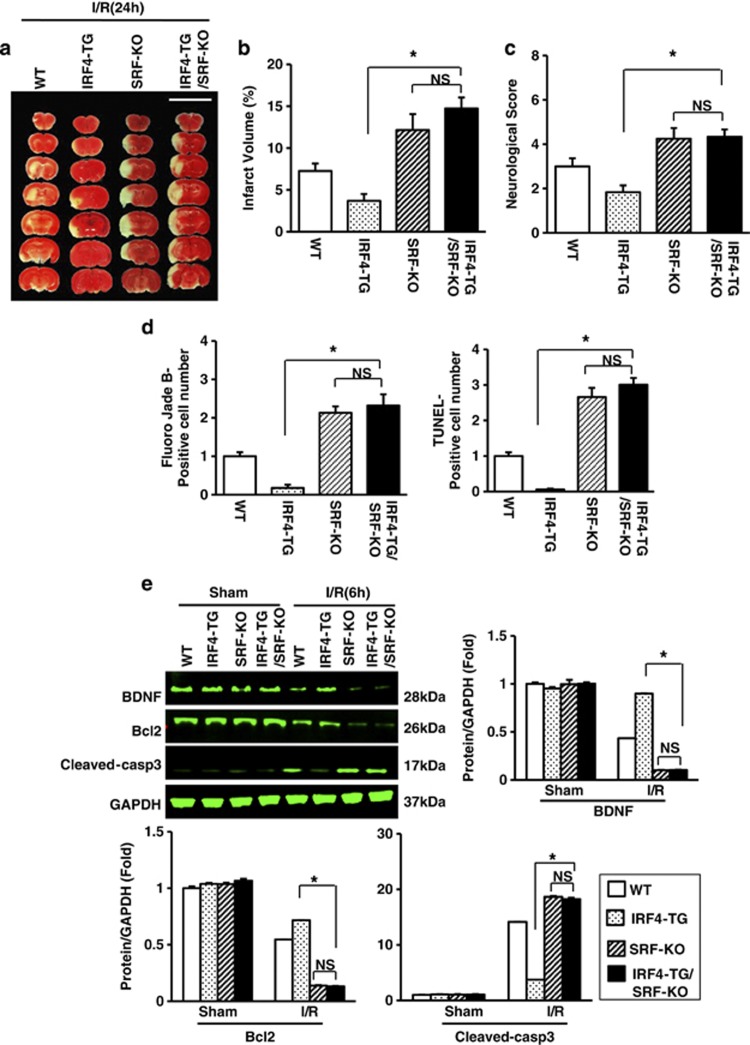
SRF-deficiency abrogates IRF4-mediated neuroprotection *in vivo*. (**a**–**c**) SRF ablation reversed cerebroprotection upon IRF4 overexpression. (**a**) TTC-stained brains from IRF4-TG, SRF-KO and IRF4nTG/SRF-KO mice 24 h after I/R. Scale bar: 10 mm. (**b** and **c**) Quantification of infarct volumes (**b**) and neurological scores (**c**) 24 and 72 h after I/R. Four to six independent experiments were performed. (**d**) IRF4-TG, SRF-KO and IRF4nTG/SRF-KO mice were subjected to 24-h I/R. The Fluoro-jade B- (left panel) and TUNEL-positive (right panel) neurons in brains were quantified. Three to four independent experiments were performed. (**e**) Immunoblotting and quantification of the BDNF, Bcl2 and cleaved caspase-3 levels in the brains of IRF4-TG, SRF-KO or IRF4nTG/SRF-KO mice subjected to a sham operation or 6-h I/R. GAPDH served as a loading control. Three independent experiments were performed. For **b**–**e**, *P<0.0001 compared with IRF4-TG mice. NS: not significant, *post hoc* Tukey's test with Bonferroni correction. The values are the means±S.E

We further investigated whether SRF is a requisite for IRF4-dependent survival. Neuron-specific SRF-knockout mice (SRF-KO), similar to IRF4-KO mice (Supplementary Figure 6a), were generated (which complied with Mendel's ratio) and were active and fertile. The IRF4-TG mice were then crossed with SRF-KO mice to generate IRF4-TG/SRF-KO mice (Supplementary Figure 6a), followed by validation via western blotting (Supplementary Figure 6b). Notably, SRF does not affect IRF4 expression either before or after I/R (Supplementary Figure 6c). As shown in Figure 7a and quantified in Figure 7b, the IRF4-TG/SRF-KO mice displayed an approximately fourfold increase in infarct volume compared with the IRF4-TG mice; however, the infarct volume of the IRF4-TG/SRF-KO mice was comparable with that of the SRF-KO mice, further supporting the notion that SRF is the primary target of IRF4 in cerebral ischaemia. Accordingly, the neurological deficit was also more severe in the IRF4-TG/SRF-KO mice than in the IRF4-TG mice (Figure 7c). In this regard, the IRF4-TG/SRF-KO mice again showed no significant difference from the SRF-KO mice (Figure 7c). In addition, the protective effect mediated by IRF4 overexpression was completely abolished when SRF was deleted (Figures 7d and e), demonstrating that the protective effects of IRF4 depend on the presence of SRF. Collectively, our data demonstrated that SRF is necessary for IRF4 to exert a neuroprotective effect.

## Discussion

Neuroprotection depends on the expression of endogenous protective programmes that are regulated by transcription factors.^6^ Thus, the identification of such new modulators and the elucidation of their regulation are highly warranted. Despite a pro-survival role in oncogenesis, whether IRF4 orchestrates cell fate in normal cells has hitherto been unknown. In the present study, we for the first time depicted IRF4 as a critical neuroprotective regulator during stroke. In MCAO-challenge mice, we readily detected IRF4 expression in neurons, which was upregulated by I/R insults. Using genetic manipulations, we showed that IRF4-TG mice displayed reduced infarct lesions and improved neurological deficits; conversely, IRF4 deficiency rendered the mice more prone to I/R-induced brain damage and neurological disorders. IRF4 rescued neurons from death and apoptosis both *in vivo* and *in vitro*. Mechanistically, we demonstrated that following stroke, IRF4 binds to the promoter region of SRF, thereby initiating SRF transcription. Furthermore, in primary cortical neurons, SRF was both necessary and sufficient for IRF4-mediated neuronal survival. Thus, we speculated that in response to stroke, IRF4 upregulates SRF to counteract programmed neuronal death. The SRF-TG mice displayed profound cerebroprotective effects compared with those of the IRF4-TG mice. More importantly, the protective effect of IRF4 was completely abolished in SRF-KO mice but restored in SRF-TG mice despite the deletion of IRF4. Therefore, we showed that IRF4-SRF was a novel neuroprotective pathway in ischaemic stroke with potential therapeutic applications.

IRF4 was initially characterised as an immune system-restricted protein.^15, 24^ Nonetheless, we and others have previously demonstrated the presence of IRF4 in the brain as well as in other organs and tissues.^13, 25^ Unfortunately, the biological function of IRF4 in neurological diseases remains largely unknown. Herein, although the underlying mechanism is unclear, we have shown that IRF4 can be specifically induced by I/R both *in vivo* and *in vitro*. Intriguingly, Grumont *et al.* reported that IRF4 expression can be induced by NF-*κ*B following mitogenic stimulus, which is also activated in experimental stroke.^26, 27^ Thus, IRF4 was likely induced by factors such as NF-*κ*B to combat these detrimental events. For example, IRF4 expression can also be strongly upregulated in B cells by interleukin-4.^28^ It should be also noted that IRF4 *per se* has the potential to regulate its own transcription, at least partially, in terminally differentiated plasma cells.^16, 29^ However, in contrast to other IRFs, IRF4 is not IFN responsive. Hence, an investigation into the mechanism of the post-stroke induction of IRF4 is required in future studies.

IRF4 has been shown as a requisite for the survival of malignant cells, particularly many lymphoid malignancies. Shaffer *et al.* recently showed that myeloma cells are addicted to IRF4 expression.^16^ Even a modest reduction of IRF4 culminates in significant cell death. Nevertheless, whether IRF4 has a broader role in cell survival than previously proposed is not known. The present study elucidated a neuronal role of IRF4 in which IRF4 is both sufficient and necessary for neuronal survival in experimental stroke. Furthermore, we deciphered the underlying molecular basis for the protective effects of IRF4. IRF4 consists of a C-terminal IRF association domain and a highly conserved N-terminal DNA-binding domain characterised by five tryptophan residues. IRF4 could function either as a positive or negative transcriptional regulator depending on the DNA-binding domain in the promoter of target genes.^24, 30^ We recognised the IRF4-binding site in the SRF promoter region, which was further confirmed via a luciferase reporter assay using a mutant SRF promoter. In line with our data, for many genes in the immune system, IRF4 acts as a transcription activator by binding to the composite DNA element termed IFN-stimulated response element.^31, 32^ After the binding, IRF4 endows neurons with the ability to activate an SRF-dependent profile and thus a sequential resistance towards I/R-induced neuronal death. Notably, our data conflict with a previous finding that SRF mediates activity-induced gene expression and synaptic plasticity but not neuronal viability.^21^ However, in their study, Ramanan *et al.* reported that SRF-deficient adult neurons showed a normal morphology that does not affect neuronal survival at the basal level; this finding is consistent with our finding that SRF gene manipulation does not affect cell fate in the absence of an OGD challenge. Accordingly, we discovered that either SRF-overexpression or -KO does not impact neuronal death or the expressions of pro-survival BNDF and Bcl2- or pro-apoptotic-cleaved caspase-3. In agreement with this notion, Rieker *et al.* showed that the deletion of SRF-rendered dopaminergic neurons more vulnerable to MPTP-induced oxidative stress.^33^ In addition, Chang *et al.* showed that SRF is essential for the BDNF-induced neuroprotection of cortical neurons in response to trophic deprivation and DNA damage.^7^ Thus, the IRF4-SRF signalling pathway is neuroprotective *in vivo*, at least during stroke. Whether SRF potentiates neuronal survival depends on the appropriate experimental setting, stimulus and (more importantly) expression level, as regulated by upstream transcription factors. Using a cell survival assay, we demonstrated that SRF sufficiently recapitulated IRF4-mediated neuroprotection against an OGD challenge. Concurrently, ectopically expressed IRF4 failed to complement the SRF deficiency; this finding supports the notion that IRF4 functions upstream of SRF. Thus, SRF is a *bonna fide* target of IRF4 after neuronal I/R injury.

In conclusion, our data delineate a previously unrecognised pro-survival pathway after ischaemic stroke: IRF4 initiates SRF transcription in neurons, which in turn promotes the expression of a myriad of SRF-dependent neuroprotective factors. Superimposed on this biological signalling pathway are the enhanced neuronal survival and reduced infarct lesions observed in IRF4- and SRF-TG mice. Furthermore, we showed that, at least during I/R injury, IRF4-mediated protection is largely SRF dependent. These findings indicate that IRF4-SRF may be a potential therapeutic target for stroke treatment and the prevention of neurological disorders.

## Materials and Methods

### Animals

All experimental protocols using animals were approved by the Animal Care and Use Committee of Renmin Hospital of Wuhan University. Neuron-specific Cre transgenic mice (CaMKII*α*-Cre; Stock No. 005359) and IRF4 conditional (floxed) mutant mice (Irf4^fl/fl^; Stock No. 009380) were purchased from the Jackson Laboratory (Bar Harbor, ME, USA). Genotyping of mouse tail digests was performed using the following primers: CaMKII*α*-Cre forward: 3′-GCGGTCTGGCAGTAAAAACTATC-5′ CaMKII*α*-Cre reverse: 3′-GTGAAACAGCATTGCTGTCACTT-5′ Irf4^fl/fl^ forward: 3′-TGCCTTTGGGACGGATGCTC-5′ Irf4^fl/fl^ reverse 1: 3′-CTTCTAGCTGACCACTAAGAAC-5′ (for WT); Irf4^fl/fl^ reverse 2: 3′-GACCACTACCAGCAGAACAC-5′ (for mutant). Neuron-specific SRF-KO mice were generated similarly by mating SRF conditional (floxed) mutant mice (Srf^fl/fl^; Stock No. 006658; Jackson Laboratory) with CaMKII*α*-Cre mice. Primers used for PCR genotyping of SRF-KO mice were: Srf^fl/fl^ forward primer: 3′-TGCTTACTGGAAAGCTCATGG-5′ Srf^fl/fl^ reverse: 3′-TGCTGGTTTGGCATCAACT-5′. Full-length mouse IRF4 cDNA was reverse-transcribed using primers: forward: 3′-CCAGATTACGCTGATAACTTGGAGACGGGCAGCCG-5′ reverse: 3′-AGGGAAGATCTTGATTCACTCTTGGATGGAAGAAT-5′ and then inserted into the pCAG-CAT promoter expressing the chloramphenicol acetyltransferase (CAT) gene floxed under the CAG promoter by ligation-independent cloning. Irf4-floxed mice were produced by microinjecting the construct into fertilised embryos (C57BL/6 background). We then crossed the mice with a second transgenic mice, in which a transgene-encoding Cre recombinase was driven by the neuron-specific CaMKII*α* promoter (CaMKII*α*-Cre mice). All mice were housed in an environment with controlled light (12 h light/12 h dark), temperature and humidity, with food and water available *ad libitum*.

### Induction of focal cerebral ischaemia

Male mice aged 11–12 weeks (25–30 g) were subjected to cerebral ischaemia produced by occluding the left MCA using the intraluminal filament technique, as described in previous studies.^34, 35, 36, 37, 38^ Briefly, after anaesthetisation with 2.5–3% isoflurane in O_2_, the mice were placed on a heating plate to maintain a rectal temperature of 37±0.5 °C. MCAO was achieved by introducing a 6-0 silicon-coated monofilament (Doccol, Redland, CA, USA) into the internal carotid artery through an incision in the left common carotid artery. The filament was then advanced into the cerebral arterial circle to obstruct the origin of the MCA. A decreased regional cerebral blood flow (rCBF) and its restoration were confirmed with Doppler analysis (Periflux System 5010; Perimed, Järfälla, Sweden). After 45 min of MCAO, the filament was withdrawn. rCBF was restored for the durations indicated in the text. The animals were returned to a heated cage for 2 h to recover, with free access to food and water. A similar procedure was performed in the sham controls, but the filament was withdrawn immediately after the rCBF diminished. The blood gases, systolic blood pressure, diastolic blood pressure and heart rate were recorded in randomly selected conscious mice.

### Indian ink staining

Indian ink staining was performed to confirm that the cerebral vasculature of the experimental mice was intact, as described in previous studies.^34^ Briefly, after anaesthesia, the mice were transcardially perfused with 2 ml of room temperature staining solution containing 10% (W/V) gelatine (Amresco, Solon, OH, USA) and 50% (V/V) Indian ink (Solarbio, Beijing, China), which was ceased when their tongues, lips and gums turned black. After anaesthetisation with 2.5–3% isoflurane in O_2_, the brains were rapidly isolated and immersed in 10% buffered formalin for 24 h. The integrity of the circle of Willis and their branches were visualised using a Nikon D700 digital camera (Nikon, Tokyo, Japan).

### Neurological deficit scores

Twenty-four and seventy-two hours after the MCAO induction, neurological deficits were assessed using a nine-point scale.^35, 39^ The scale was based on the following observations: (1) absence of neurological deficits (0 points); (2) left forelimb flexion upon suspension by the tail or failure to fully extend the right forepaw (1 point); (3) left shoulder adduction upon suspension by the tail (2 points); (4) reduced resistance to a lateral push towards the left (3 points); (5) spontaneous movement in all directions with circling to the left only if pulled by the tail (4 points); (6) circling or walking spontaneously only to the left (5 points); (7) walking only when stimulated (6 points); (8) no response to stimulation (7 points); and (9) stroke-related death (8 points).

### Infarct volume determination

The brains were removed 24 and 72 h after MCAO, cut into seven 1-mm coronal sections, and then immersed in a 2% 2,3,5-triphenyl-2H-tetrazolium chloride solution for 15 min at 37 °C. A red colour indicated normal brain tissue, whereas a pale grey colour indicated infarcted tissue. The sections were photographed and analysed using Image-Pro Plus 6.0 (Media Cybernetics, Bethesda, MD, USA). The infarct volume (%) of the seven slices was calculated after correcting for oedema, as previously described.^35, 36, 37^

### MRI

MRI was performed using a 7.0-T magnetic resonance scanner (BRUKER, BioSpec 70/20USR, Billerica, MA, USA). The mice were anaesthetised with chloral hydrate (Sigma, 15307-500G-R, St. Louis, MO, USA). We used a volume coil for the radiofrequency transmitter and a surface coil for the receiver. The temperature was maintained via a heating block built into the gradient system. We monitored respiration throughout the entire scan. We acquired images with a two-dimensional T2-weighted fast spin echo sequence with the following parameters: slice thickness 0.50 mm, FOV (field of view) 2 cm, echo time/repetition time=26.7/2000 ms, resolution=0.078 × 0.078 mm, echo train length=4, number of averages=4, matrix size=256 × 256. The infarct volume, expressed as a percentage of the contralateral hemisphere, was summed (a total of five slices), multiplied by the slice thickness and corrected for oedema.

### Immunohistology

The mice were anaesthetised and transcardially perfused with 0.1 M sodium phosphate buffer (pH 7.4) followed by 4% paraformaldehyde in phosphate buffer for 15 min. Immunocytochemistry was performed as previously described.^34, 35, 36^ Briefly, the brains were fixed in the same paraformaldehyde solution for 6–8 h at room temperature. After immersion overnight at 4 °C in phosphate buffer containing 30% sucrose, the brains were embedded in OCT solution. The OCT-embedded brains were cut into 5-*μ*m-thick sections according to standard procedures. In accordance, primary neurons cultured on coverslips were fixed in ice-cold acetone. The cryosections or coverslips were then washed in PBS containing 10% goat serum and incubated overnight at 4 °C with the following primary antibodies: mouse anti-NeuN (MAB377; 1 : 200; Millipore, Temecula, CA, USA), chicken anti-MAP2 (Ab5392; 1 : 100; Abcam, Cambridge, UK), rabbit anti-IRF4 (sc28696; 1 : 50, Santa Cruz Biotechnology Inc., Santa Cruz, CA, USA), rabbit anti-cleaved caspase-3 (#9661; 1 : 100; Cell Signaling Technology, Danvers, MA, USA), rabbit anti-SRF (sc13029, 1 : 100, Santa Cruz Biotechnology Inc.), rabbit anti-BDNF (ab72439, 1 : 50, Abcam) and rabbit anti-Bcl2 (#2870; 1 : 50; Cell Signaling Technology). After being washed in PBS, the sections were incubated with the indicated secondary antibodies for 1 h. The following secondary antibodies were used: goat anti-chicken IgY (H&L, DyLight 488, ab96947; Abcam), goat anti-mouse IgG Alexa Fluor 568 conjugate (A11004; Invitrogen, Carlsbad, CA, USA) and anti-rabbit IgG Alexa Fluor 568 conjugate (A11011; Invitrogen). The nuclei were stained with 4′6-diamino-2-phenylindole (S36939, Invitrogen). For the TUNEL assay, the sections were tested for neuronal apoptosis with an ApopTag Plus *In Situ* Apoptosis Fluorescein Detection Kit (S7111; Millipore) according to the manufacturer's protocol. The TUNEL-positive cells were evaluated using a microscope and quantified under high-power magnification ( × 200). Fluoro-Jade B staining was also used to detect I/R-induced neuronal degeneration following the manufacturer's instructions. Briefly, the brain sections were immersed in a solution containing 1% NaOH and 80% alcohol for 5 min, followed by 70% alcohol for 2 min and washed with distilled water for 2 min. The sections were then incubated in a fluorescent Fluoro-Jade B (AG310, Millipore, Billerica, MA, USA) stain diluted in PBS as recommended by the manufacturer.

### Image acquisition and analysis

Images were visualised under a fluorescence microscope OLYMPUS DX51 (Olympus, Tokyo, Japan) using DP2-BSW Ver. 2.2 software (Olympus, Tokyo, Japan), and image analysis was performed with Image-Pro Plus 6.0 software. The analysis was performed by two researchers blinded to experimental groups. All images were evaluated under × 20 magnification except Figure 1e ( × 40 magnification). Three to four visual fields in both ipsilateral and contralateral were randomly selected from each coverslip. In Figures 1b and c, cells co-stained with IRF4 and NeuN or MAP2 from both peri-infarct regions in cortex and corresponding regions of the contralateral hemisphere were counted, respectively. In Figure 1d, cortex, striatum and hippocampus in bilateral hemispheres were analysed, respectively. Neurons with IRF4 staining were considered positive. In Figure 1e, the integrated optical density of IRF4 in primary cortical neurons were quantified at different time points. In Figure 4a, Fluoro Jade B- and TUNEL-positive cells (with 4′6-diamino-2-phenylindole) in peri-infarct regions of cortex were counted. In Figures 4c, 6d, h and i, NeuN (in peri-infarct regions of cortex) co-stained with cleaved-casp3, SRF, BDNF and Bcl2 were included in evaluation, respectively. We calculated the fold changes of positive cells compared with contralateral sides, WT or NTG mice.

### Tissue preparation

For quantitative real-time PCR and western blot analysis, the mice were anaesthetised and transcardially perfused with cold sodium phosphate; the brains were then immediately removed. To collect the tissue in an unbiased manner that reflected the global extent of the infarcts, the olfactory bulbs and 1-mm sections of the anterior and posterior brain tissue were excised. We then collected the remaining tissues of the ipsilateral (including the infarct and peri-infarct areas) and contralateral (normal) hemispheres. All tissues were immediately frozen in liquid nitrogen and stored at −80 °C.

### Quantitative real-time PCR

Total RNA was extracted using TRIZOL reagent (Invitrogen) following the manufacturer's protocol and was reverse-transcribed into cDNA using a Transcriptor First Strand cDNA Synthesis Kit (Roche, Indianapolis, IN, USA). Quantitative RT-PCR analysis was performed as previously described with the LightCycler 480 SYBR Green 1 Master Mix (Roche) and the LightCycler 480 QPCR System (Roche). The PCR conditions were as follows: 95 °C for 10 min; 40 cycles of 95 °C for 10 s, 60 °C for 10 s and 72 °C for 20 s; and a final extension at 72 °C for 10 min. The relative quantity of the mRNAs was calculated after being normalised to GAPDH. The primer pairs used are as listed in Supplementary Table 2.

### Western blot analysis

Western blotting was performed as previously described.^35, 36^ Briefly, lysates were prepared from brains or cultured cells via incubation in lysis buffer. Samples containing 50 *μ*g protein per well were separated on 8–12% SDS-PAGE gels and transferred onto PVDF membranes (Millipore, Bedford, MA, USA). The membranes were incubated in blocking buffer (TBST containing 5% skim milk powder) for 1 h at room temperature and immersed in primary antibodies overnight at 4 °C. The membranes were washed and subsequently incubated with the secondary antibodies (goat anti-rabbit IRDye 800CW, 926-32211, goat anti-mouse IRDye 800CW, 926-32210 or donkey anti-goat IRDye 800CW, 926-32214; LI-COR Biosciences, Lincoln, NE, USA) for 1 h at room temperature. Finally, an Odyssey infrared imaging system (LI-COR Biosciences) was employed to detect the blot signals; signals were quantified using an Odyssey software (LI-COR).The primary antibodies used were as follows: rabbit anti-cleaved caspase-3 (Asp 175; #9661), anti-fosB (#2251), anti-Bcl2 (#2870), anti-Bax (#2772), anti-PSD95 (#2507), anti-EGR1 (#4153) and anti-Cdk5 (#2680; all from Cell Signaling Technology, Beverly, MA, USA; 1 : 1000); mouse anti-GAPDH (MB001; both from Bioworld Technology, Minneapolis, MN, USA); rabbit anti-BDNF (ab72439; from Abcam); and rabbit anti-IRF4 (sc28696), anti-SRF (H300; sc13029), anti-JunB (sc46), anti-Gelsolin (H-70, sc48769), anti-Egr-2 (H-220, sc20690), anti-Nur77 (M-210, sc5569) and goat anti-CTGF (L-20, sc14939; all from Santa Cruz Biotechnology). Mouse anti-GAPDH served as the internal control.

### Cell culture and *in vitro* manipulation

The primary cortical neurons were prepared from Sprague–Dawley rat brains within 1 day of birth or neocortices from fetal mice (embryonic day 14–15). Briefly, the cortices were dissociated via incubation for 15 min at 37 °C in 2 ml of 0.125% trypsin (GIBCO, Grand Island, NY, USA), followed by the addition of 4 ml of Dulbecco's modified Eagle's medium/F-12 (GIBCO) containing 20% fetal bovine serum (GIBCO) to inactivate trypsin. The cell suspension was centrifuged for 10 min at 180 × *g* and resuspended in Dulbecco's modified Eagle's medium containing 20% fetal bovine serum. The cells were passed through 200-*μ*m sterile filtres and seeded on plates coated with poly-L-lysine (10 mg/ml, Sigma). The neurons were cultured in Neurobasal Medium (GIBCO) fortified with B27 (GIBCO) for 24 h at 37 °C and 5% CO_2_. AraC (10 *μ*M, Sigma) was added to the medium 24 h after plating to inhibit cell proliferation, and the medium was changed every 48 h. The cells were cultured for 5 days before the experiments. To model the I/R conditions *in vitro*, the neuronal cultures were exposed to transient oxygen and glucose deprivation (OGD) for 60 min and then returned to normal culture conditions for various periods. For the OGD, the neurobasal medium was replaced with serum-free, glucose-free Locke's buffer (154 mM NaCl, 5.6 mM KCl, 2.3 mM CaCl_2_, 1 mM MgCl_2_, 3.6 mM NaHCO_3_, 5 mM HEPES and 5 mg/ml gentamicin, pH 7.2), and the cultures were incubated in an experimental hypoxia chamber in a saturated atmosphere of 95% N_2_ and 5% CO_2_. The control cells were cultured in the presence of normal levels of glucose and were incubated for the same periods in a humidified atmosphere of 95% air and 5% CO_2_.

### Recombinant adenoviral vectors

We constructed adenoviruses carrying sequences encoding mouse IRF4 (AdIRF4), SRF (AdSRF) and short hairpin RNA-targeting IRF4 (shIRF4) or SRF (shSRF) for the *in vitro* studies. AdIRF4 and AdshIRF4 were generated and described previously;^13^ AdSRF was purchased from ABM Inc (Accession number: NM_020493, ABM, Richmond, BC, USA). For special knockdown of the expression of SRF, SureSilencing shRNA Plasmids for SRF (KR66778G) under the control of a U1 promoter were purchased from SABiosciences (Valencia, CA, USA). Using a similar process as AdshIRF4, the recombinant adenovirus was generated.^13^ The cultured cortical neurons were transfected with adenovirus at an MOI of 100 for 48 h.

### Cell viability

To examine cell viability, a nonradioactive cell counting kit-8 (CCK-8) assay (CK04, Dojindo, Kumamoto, Japan) was used in accordance with the manufacturer's protocol. LDH release, an indicator of cell lysis, was determined via a colorimetric LDH cytotoxicity assay (G1782, Promega, Madison, WI, USA). Three independent experiments were performed.

### ChIP

Briefly, cultured mouse primary cortical neurons were transfected with HA-IRF4 and pcDNA3.1 for 48 h and the then fixed with 1% formaldehyde, washed with ice-cold PBS, harvested and sonicated. The soluble chromatin was then immunoprecipitated using anti-IRF4 antibody (4964S, Cell Signaling Technology) or normal rabbit IgG (2729S, Cell Signaling Technology) followed by protein G-magnetic beads (10004D, Invitrogen). Precipitated DNAs were harvested by phenol/chloroform extraction and amplified using qPCR for 40 cycles. The PCR primers for ChIP assays were listed in Supplementary Table 3.

### Dual luciferase-reporter assay

Mouse SRF promoter (NC_000083.6, 46555997-46557470, negative strand) was amplified from C57/BL genome using SRF-IRF4-F/R primers. Then it was inserted into a pGL3-basic vector (Promega) between the *Kpn*I and *Hin*dIII restriction sites to generate WT SRF promoter (WT-mSRF-Luc). The IRF4-binding sites in the SRF promoter (5′-CTATGTTACTGAAACTATTTA-3′) were predicted by Genomatix MatInspector online software (http://www.genomatix.de/). The core 5′-GAAA-3′ IRF4-binding site in the P3 region of murine SRF promoter was deleted using SRF-IRF4-MF/R primers by the fusion-PCR approach. The mutated SRF promoter sequence was then inserted into pGL3-basic vector using the same restriction sites as wide type. The primary mouse cortical neurons were cultured in a 24-well plate and co-transfected with the indicated vectors for 24 h. The neuronal culture was then subjected to OGD/reperfusion for 24 h before harvesting. The cells were lysed with 100 *μ*l of passive lysis buffer (Promega) per well for 30 min at room temperature. After centrifuging, the supernatant was collected and assessed for luciferase activity using a Single-Mode SpectraMax Microplate Reader as described previously. The primers used for vector construction of luciferase reporter gene were listed in Supplementary Table 4.

### Statistical analysis

The data were expressed as the means±S.E. Differences between groups were determined using an analysis of variance, followed by a *post hoc* Tukey's test. Comparisons between two groups were performed using an unpaired Student's *t*-test. All *in vivo* and imaging studies were performed in a blinded manner. *P*<0.05 was considered statistically significant.

## Abbreviations

OGD: oxygen glucose deprivation
SRF: serum response factor
I/R: ischaemia/reperfusion
IRF: interferon regulatory factor
CaMKII: Ca2+/calmodulin-dependent protein kinase II
WT: wild-type
NTG: non-transgenic
GFP: green fluorescent protein
TUNEL: terminal deoxynucleotidyl transferase-mediated dUTP-biotin nick end labelling
MCAO: middle cerebral artery occlusion
BDNF: brain-derived neurotrophic factor
rCBF: regional cerebral blood flow
MRI: magnetic resonance imaging
IRF4-TG: IRF4 transgenic
IRF4-KO: IRF4 knockout
MCAO: middle cerebral occlusion
NeuN: neuron-specific nuclear protein
MAP-2: microtubule-associated protein-2
SRF-KO: SRF-knockout
MAC: middle cerebral artery
