# Supplementary material for: IRF4 is a novel mediator for neuronal survival in ischaemic stroke
Source: Cell Death Differ. 2014 Feb 7;21(6):888–903. doi: 10.1038/cdd.2014.9 (PMC4013523; doi:10.1038/cdd.2014.9)
Supplement: Supplementary Information [file cdd20149x1.pdf]

IRF4 is a Novel Mediator for Neuronal Survival in Ischemic Stroke

Sen Guo[1,2,*], Zuo-Zhi Li [3 *], Ding-Sheng Jiang [1,2*], Yan Yun Lu[1,2], Yi Liu[4], Lu Gao[6], Shu-Min Zhang [1,2], Hao Lei [5], Li-Hua Zhu [1,2], Xiao-Dong Zhang [4] , De-Pei Liu[3 #,] , Hongliang Li [1,2, #]

1 Department of Cardiology, Renmin Hospital of Wuhan University, Wuhan 430060, China; 2 Cardiovascular Research Institute, Wuhan University, Wuhan 430060, China; 3 State Key Laboratory of Medical Molecular Biology, Department of Biochemistry and Molecular Biology, Institute of Basic Medical Sciences, Chinese Academy of Medical Sciences & Peking Union Medical College, Beijing 100005, China; 4 College of Life Sciences, Wuhan University, Wuhan 430072, China; 5 Wuhan Center for Magnetic Resonance, State Key Laboratory of Magnetic Resonance and Atomic and Molecular Physics, Wuhan Institute of Physics and Mathematics, Chinese Academy of Sciences, Wuhan, China. 6 Department of Cardiology, Institute of Cardiovascular Disease, Union Hospital, Tongji Medical College, Huazhong University of Science and Technology, Wuhan, 430022, China. (* Sen Guo, Zuo-Zhi Li and Ding-Sheng Jiang are co-first authors.)

Correspondence and requests for materials should be addressed to H.L.L

(E-mail: lihl@whu.edu.cn)

Supplementary Figure S1-S6

Supplementary Table S1-S4

Supplementary Figure S1

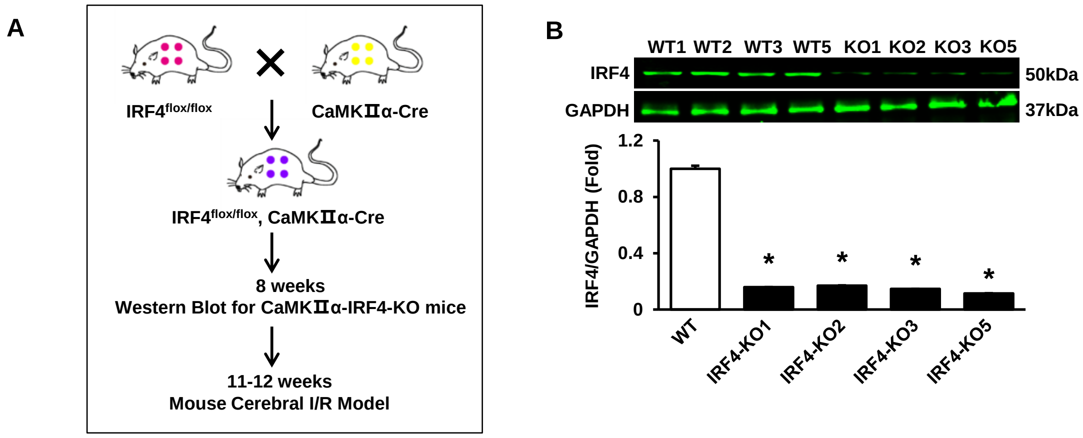

Supplementary Figure S1. Generation of neuron-specific IRF4-knockout mice. (a) Schematic representation of generation of neuron-specific IRF4-knockout (IRF4-KO) mice. **(b)** Immunoblotting of IRF4 in the brain lysates from IRF4-KO mice (left) and the relative IRF4 levels (right). *P<0.0001, <0.0001, <0.0001, <0.0001 versus wild type (WT), respectively, unpaired Student's t-test. The values are the means ± SE of three independent experiments in triplicate.

Supplementary Figure S2

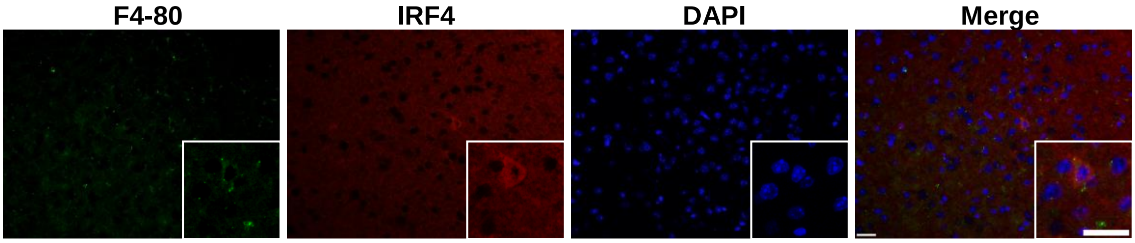

Supplementary Figure S2. IRF4 expresses at a relatively low level in microglia. Representative immunofluorescence images of IRF4 (red), F4-80 (green) and DAPI (blue) in the brains of wild-type mice out of three independent experiments. Scale bar: 20 μm.

Supplementary Figure S3

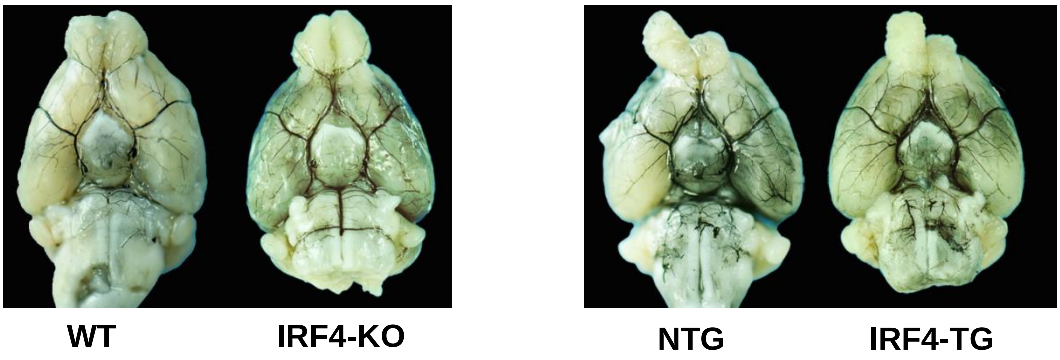

Supplementary Figure S3. Genetic manipulations of IRF4 do not impact the intergrity of cerebral vasculature. Representative images showing the integrity of the cerebral vasculature stained with India ink in WT, IRF4-KO, NTG, and IRF4-TG mice out of three independent experiments.

Supplementary Figure S4

A

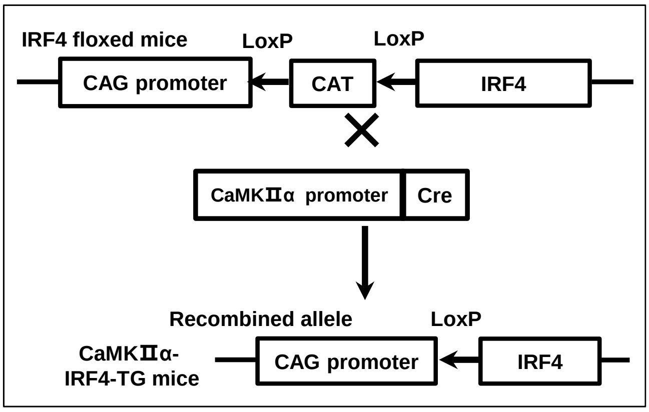

B

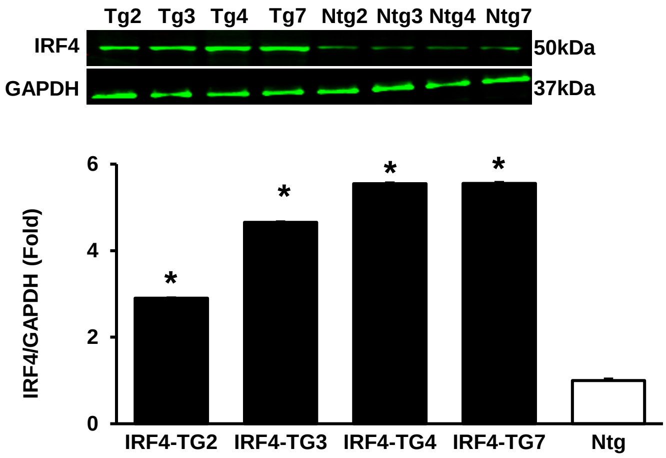

Supplementary Figure S4. Generation of neuron-specific IRF4-transgenic mice. (a) Schematic representation of generation of neuron-specific IRF4-transgenic (IRF4-TG) mice. **(b)** Immunoblotting of IRF4 in the brain lysates from IRF4-TG mice (left) and the relative IRF4 levels (right). *P<0.0001, <0.0001, <0.0001, <0.0001 compared with non-transgenic mice (NTG), respectively, unpaired Student's t-test. The values are the means ± SE of three independent experiments in triplicate.

Supplementary Figure S5

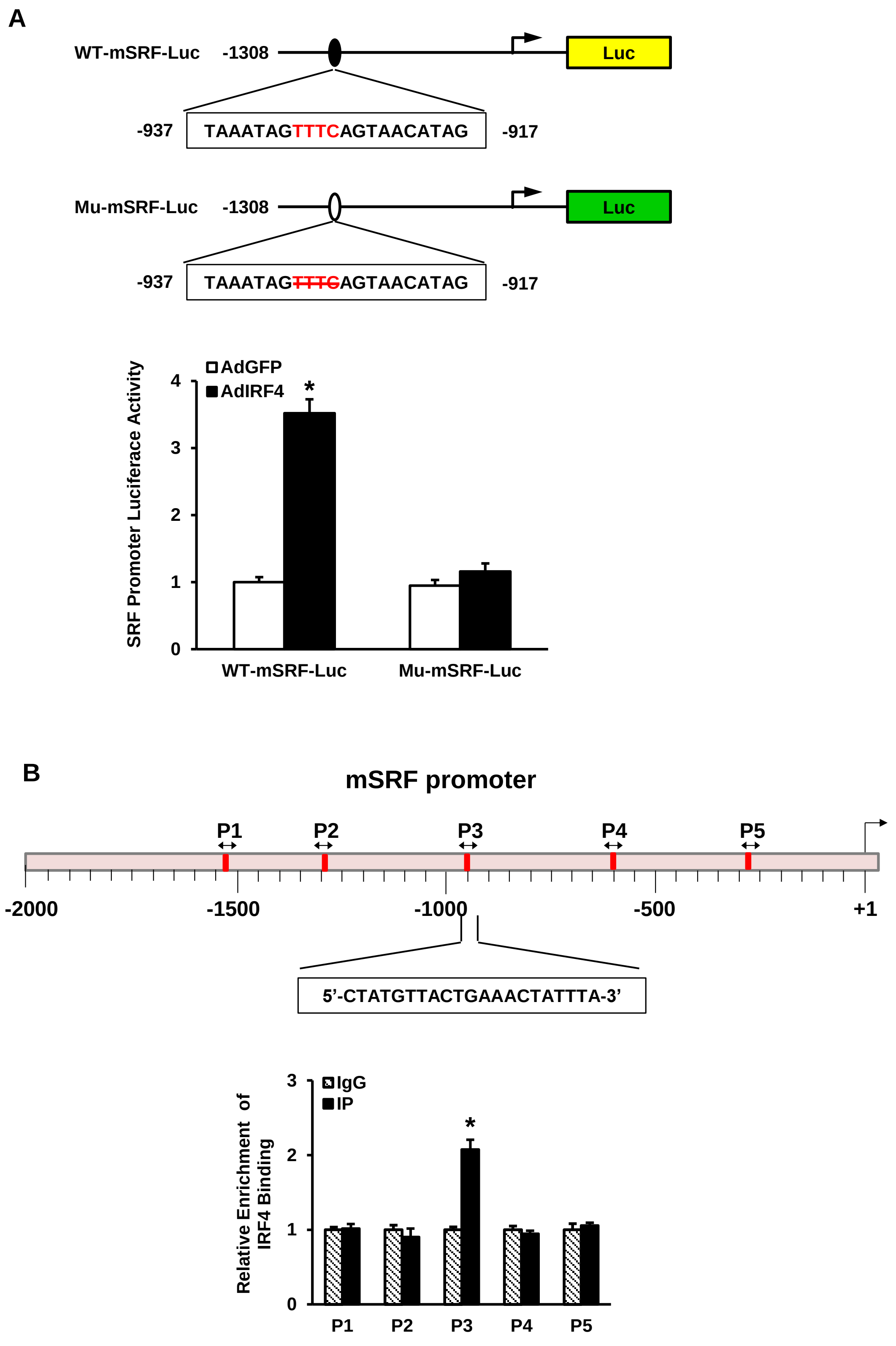

Supplementary Figure S5. IRF4 binds to the promoter region of SRF. (a) The primary mouse cortical neurons were transfected with the wild-type (WT-mSRF-Luc) or mutant SRF promoter (Mu-mSRF-Luc) and infected with AdGFP or AdIRF4 prior to the OGD treatment. The luciferase activity was assessed and quantified (bottom panel). The values are the means ± SE of three independent experiments. *P<0.0001 versus AdGFP. NS: not significant, unpaired Student's t-test. **(b)** The putative IRF4 binding sites (P3) were identified in the promoter region of the murine SRF gene (upper panel). P1, P2, P4, and P5 served as negative controls. Lower panel: IRF4 was enriched in the P3 region as shown by chromatin immunoprecipitation (ChIP). *P=0.0015 versus IgG. The values are the means ± SE of three to five independent experiments in triplicate, unpaired Student's t-test.

Supplementary Figure S6

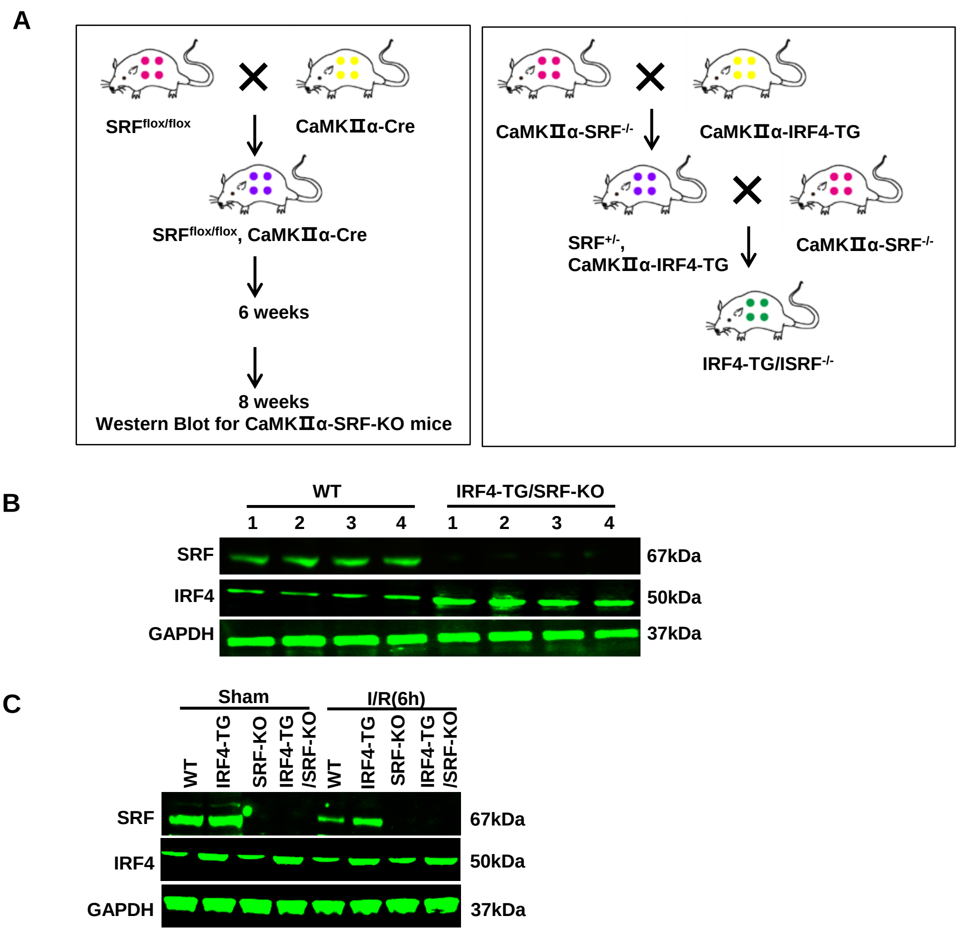

Supplementary Figure S6. Generation of neuron-specific SRF knockout (SRF-KO) mice and IRF4-TG/SRF-KO mice. (a) Schematic representation of generation of SRF-KO (left panel) and IRF4-TG/SRF-KO mice (right panel). **(b)** Immunoblotting of SRF and IRF4 in WT and IRF4-TG/SRF-KO mice. GAPDH served as a loading control. **(c)** IRF4-TG, SRF-KO and IRF4nTG/SRF-KO mice were subjected to a sham operation or 24-h I/R. Using immunoblotting, the levels of SRF and IRF4 were detected in brains prior to and after I/R. GAPDH served as a loading control.

Supplementary Table S1

	WT	IRF4-KO	NTG	IRF4-TG
**pH**	7.333±0.032	7.263±0.056	7.348±0.015	7.323±0.027
**pO_2 (mmHg)**	125.50±2.02	126.00±5.73	116.75±2.63	125.25±4.66
**pCO_2 (mmHg)**	32.25±2.92	33.25±3.45	35.50±3.43	31.50±2.25
**DBP (mmHg)**	105.98±1.78	106.59±2.15	104.40±2.15	105.41±2.68
**SBP (mmHg)**	132.73±1.90	137.64±3.17	134.72±1.57	136.24±0.96
**Heart rate (b.p.m.)**	616.08±23.46	610.92±9.03	640.69±13.06	609.64±13.31

Supplementary Table S1. Physiological variables of experimental groups before MCAO. Blood gas analysis, blood pressure, and heart rate were comparable between groups. Data represent mean ± SE from 8 mice of each genotype, post hoc Tukey's test with Bonferroni correction. WT: wild-type; KO: neuron-specific IRF4 knockout; NTG: non-transgenic; TG: neuron-specific IRF4 transgenic. SBP: systolic blood pressure; DBP: diastolic blood pressure.

Supplementary Table 2

Primers used for Real-time PCR.

Genes	Sequence 5'-3'
**SRF-FWD-A**	TTCAGCAAGAGGAAGACGGGCA
**SRF-REV-A**	AGTTTGCGGGTGGCAAAGGT
**BDNF-FWD-B**	AACGTCCACGGACAAGGCAACTT
**BDNF-REV-B**	CCAAAGGCACTTGACTGCTGAGCA
**c-fos-FWD-A**	CGGGTTTCAACGCCGACTA
**c-fos-REV-A**	TGGCACTAGAGACGGACAGAT
**fos-B-FWD-B**	AGGAACGCCTGGAGTTTGTCCT
**fos-B-REV-B**	AGCCGTCTTCCTTAGCGGATGT
**Junb-FWD-A**	CTATCGGGGTCTCAAGGGTC
**Junb-REV-A**	CTGTTGGGGACGATCAAGC
**Egr1-FWD-A**	TATGAGCACCTGACCACAGAG
**Egr1-REV-A**	GCTGGGATAACTCGTCTCCA
**Egr2-FWD-A**	TCAGTGGTTTTATGCACCAGC
**Egr2-REV-A**	GAAGCTACTCGGATACGGGAG
**Egr3-FWD-B**	TTGCCTGACAATCTGTACCCC
**Egr3-REV-B**	TAATGGGCTACCGAGTCGCT
**Cyr61-FWD-B**	CTCCAGAATCTACCAAAACGGG
**Cyr61-REV-B**	CGTCCAGGGAGTCCTTAATGC
**Ctgf -FWD-B**	AGACCTGTGGGATGGGCAT
**Ctgf -REV-B**	GCTTGGCGATTTTAGGTGTCC
**Gelsolin-FWD-A**	ACCAGCTCTGCTGCATCAGGTA
**Gelsolin-REV-A**	GCACAGGCACCAGGTCAAACTT
**Psd95-FWD-A**	ACCAGAAGAGTATAGCCGATTCG
**Psd95-REV-A**	GGTCTTGTCGTAGTCAAACAGG

**Sema3a-FWD-A**	TGGCAAAGCCTGTGCAGAATGC
**Sema3a-REV-A**	TGGTGCTGCAAGTCAGAGCAGT
**Ngf-FWD-B**	AGACTCCACTCACCCCGTG
**Ngf-REV-B**	GGCTGTGGTCTTATCTCCAAC
**Cdk5-FWD-A**	GGGAAGGCACCTATGGAACTG
**Cdk5-REV-A**	CAGCCTGACACGCTTCAGAG
**Actb-FWD-A**	GTGACGTTGACATCCGTAAAGA
**Actb-REV-A**	GCCGGACTCATCGTACTCC
**Actg2-FWD-A**	TGAGATGGCCACAGCAGCTTCA
**Actg2-REV-A**	CCATGCCAATGAAGGAAGGCTGG
**Npas4-FWD-A**	TGCTCTGGATGCTGATCGCCTT
**Npas4-REV-A**	AGCGGTGAACACAGGGTTTCCT
**Nur77-FWD-B**	CTCCCCGAGCCAGACTTATG
**Nur77-REV-B**	TGTCACGGTTCGGAGAGGT
**BCL2-FWD-B**	CGTCGTGACTTCGCAGAGAT
**BCL2-REV-B**	TGACATCTCCCTGTTGACGC
**GAPDH-FWD-A**	ACTCCACTCACGGCAAATTC
**GAPDH-REV-A**	TCTCCATGGTGGTGAAGACA

Supplementary Table S3

Primers used for ChIP assays

**Genes**	**Sequence 5'-3'**
**m-SRF-F1**	TCCACATTGCTGTTCATCAC
**m-SRF-R1**	GCTGGTAGTCCTGGGTTCTA
**m-SRF-F2**	GCTCCTTCCTCTCTGATGACT
**m-SRF-R2**	CACACAAACAAGTGACCTGGT
**m-SRF-F3**	CAATCCTCCTGTCTCAACCTC
**m-SRF-R3**	GAGTCTGAATCTTTGCTCCAAG
**m-SRF-F4**	GGATGATGAACGATGTGACC
**m-SRF-R4**	AGGGCAGGGATAGATTGGA
**m-SRF-F5**	CAACACTCCACTACTCTCCAAC
**m-SRF-R5**	CATAGACATACCGAACTCGCT

Supplementary Table S4

Primers used for vector construction in luciferase reporter gene assays.

Genes	Sequence 5'-3'
**SRF-IRF4-F**	GTTGGTACCTCAAGTTGATGCCAAACCAG
**SRF-IRF4-R**	GACAAGCTTCTCACTTTCCTGCCCCTATG
**SRF-IRF-MF**	GCCAGATTTAAATAGAGTAACATAGAAGTGCAAGA
**SRF-IRF-MR**	ACTTCTATGTTACTCTATTTAAATCTGGCCGGACG
